# Alarming Increase of Azole-Resistant *Candida* Causing Blood Stream Infections in Oncology Patients in Egypt

**DOI:** 10.1007/s00284-023-03468-w

**Published:** 2023-10-05

**Authors:** Hadir A. El-Mahallawy, Nesma E. Abdelfattah, Mona A. Wassef, Rasha M. Abdel-Hamid

**Affiliations:** 1 Clinical Pathology Department, National Cancer Institute, Cairo University, Cairo, Egypt; 2 Clinical & Chemical Pathology Department, Faculty of Medicine, Cairo University, Cairo, Egypt

## Abstract

Candidemia is a life-threatening invasive fungal infection in immunocompromised patients. The widespread use of azoles and the shift toward non-albicans *Candida* (NAC) species remarkably increase azole resistance in developing countries. We aimed to study candidemia trends and associated risk factors in oncology patients since they vary geographically, and rapid and appropriate treatment improves outcomes. Vitek 2 was used to identify the *Candida* species, and the E-test determined their susceptibility to azoles. *Candida* was the cause of 3.1% (*n* = 53/1701) of bloodstream infections (BSIs) during a 1-year study. *Candida tropicalis* was the most predominant species among the 30 candidemia episodes studied (36.7%), followed by *C. albicans* (33.3%). However, *C. krusei*, *C. guilliermondii*, *C. pelliculosa*, *C. parapsilosis*, *C. famata*, and *C. inconspicua* accounted for 30.0% of the isolates. An increased risk of NAC BSI was significantly associated with chemotherapy and leucopenia (*P* = 0.036 and 0.016, respectively). However, the multivariable analysis revealed that leucopenia was the only independent risk factor (*P* = 0.048). Fluconazole and voriconazole resistance were 58.3% and 16.7%, with NAC species showing higher resistance rates than *C. albicans*. Both fluconazole and voriconazole minimum inhibitory concentration (MIC) median values were higher in NAC than in *C. albicans*, but only voriconazole was significantly higher (0.220 versus 0.048 μg/ml, *P* = 0.047). In conclusion, the increased prevalence of NAC BSIs and incredibly high fluconazole resistance rates in cancer patients emphasize the necessity of antifungal stewardship to preserve voriconazole effectiveness, continued surveillance of candidemia, and future studies into azole resistance molecular mechanisms.

**Supplementary Information:**

The online version contains supplementary material available at 10.1007/s00284-023-03468-w.

## Introduction

*Candida* bloodstream infections (BSIs) have emerged as a global cause of invasive fungal infection in critically ill patients in healthcare settings. Death rates caused by this life-threatening infection range from 35 to 53% and have been linked to higher morbidity and hospital costs [1]. The spectrum of *Candida* species and antifungal susceptibility patterns vary geographically and are influenced by patients' underlying conditions. As a result, determining the local candidemia pathogenic spectrum and susceptibility trends is critical for deciding antifungal empirical treatment [2].

Several factors, including exposure to chemotherapy and broad-spectrum antibiotics, invasive interventions, extremes of age, and neutropenia, have been associated with an increased incidence of candidemia in immunocompromised patients. Positive blood cultures are traditionally considered the gold standard for diagnosing candidemia. However, underdiagnosis attributed to false negative results, in addition to the long time needed for diagnosis and the fact that prompt, appropriate antifungal treatment impacts patient outcomes, forces physicians to make a diagnosis based on clinical symptoms and the presence of potential risk factors [3]. Understanding the demographic and host factors linked to candidemia in cancer patients improves their management.

Over the past two decades, increasing azole resistance and poor susceptibility trends to commonly used antifungals have created substantial clinical concerns globally. Azoles are the most widely utilized antifungals, especially in developing countries. Fluconazole resistance is prevalent in *Candida glabrata*, *C. parapsilosis*, and *C. auris*, in addition to intrinsic resistance in *C. krusei*. Although resistance to other azoles is uncommon, several organizations have documented triazole cross-resistance [4]. As a result, antifungal susceptibility testing is essential for the management of patients and also for identifying the problem of antifungal resistance in epidemiologic studies. In addition to the selection pressure generated by antifungal use, the recent global shift toward non-albicans *Candida* (NAC) species contributes to this remarkable resistance [2, 5]. Furthermore, NAC-infected candidemia patients' clinical characteristics, related risk factors, and outcomes vary among healthcare settings [6]. Consequently, this study aimed to investigate the epidemiology, *Candida* species distribution, and antifungal susceptibility trends in cancer patients with candidemia.

## Materials and Methods

This study was conducted at the National Cancer Institute (NCI), a tertiary university hospital. It is one of Egypt's most prominent cancer centers. During 1 year, blood culture specimens referred to the Microbiology Laboratory from cancer patients receiving their therapy at NCI were analyzed. The detection of *Candida* species in at least one positive blood culture in a patient who exhibited infection-related signs and symptoms was considered *Candida* BSI [7]. One specimen was included from a patient who had multiple episodes of candidemia. Blood specimens from outpatients or patients with missing or incomplete data were excluded.

The BACTEC fluorescence series device (BACTEC 9120) was used to test the presence of BSIs in febrile cancer patients. Then positive vials were subcultured on blood, MacConkey, and Sabouraud dextrose agar (SDA) plates. *Candida* was initially identified on SDA by microscopic inspection and colony morphology. The Vitek 2 system (BioMérieux, Marcy-l'Etoile, France), CHROMagar (Becton Dickinson GmbH), and multiplex semi-nested PCR (sn-PCR) were used for further species identification.

The QIAamp DNA Mini Kits (QIAGEN, Hilden, Germany) were used for DNA extraction. The internal transcribed spacers 1 and 4 (ITS1 and ITS4) outer primers were initially used for PCR amplification. Then, the inner primers for *C. krusei*, *C. glabrata*, *C. albicans*, *C. parapsilosis*, and *C. tropicalis* were used, following the protocol of [8]. The sequences of the primers used, and the amplicon sizes are reported (Supplementary Table A1). The nested PCR outputs were then detected by 2% agarose gel electrophoresis and visualized using an ultraviolet transilluminator.

Fluconazole and voriconazole susceptibility tests were done using the E-test diffusion method, as instructed by the manufacturer (AB Biodisk). The interpretation of MIC readings was according to the Clinical Laboratory Standards Institute (CLSI) clinical breakpoints (CBPs) (CLSI, M27M44S-Ed3) and presented as sensitive, intermediate, or susceptible dose-dependent (SDD) and resistant [9]. The MIC, MIC_50_, and MIC_90_ values represent the lowest concentrations of antifungal agents required to inhibit 100%, 50%, and 90% of the isolates, respectively. The formulae MIC_50_ = number of isolates × 0.5 and MIC_90_ = number of isolates × 0.9 were used to estimate the number of inhibited isolates for each species. The antifungal MIC value inhibiting that number of isolates was then identified as MIC_50_ and MIC_90_ [10]. Wild-type (WT) phenotypes were distinguished from non-wild-type (NWT) phenotypes by using epidemiologic cutoff values (ECVs) published in CLSI, M57S-Ed4 [11]. ECVs can discriminate *Candida* isolates with and without acquired mutational resistance (non-WT) rather than sensitive and resistant isolates based on CBPs [12].

The demographic data, clinical characteristics of infected patients, and laboratory findings were collected. Leucopenia was defined as a total leucocyte count (TLC) less than 4.0 × 10^3^/μl [13] and neutropenia as an absolute neutrophil count (ANC) less than 1.0 × 10^3^/μl [14]. Inappropriate antifungal therapy was considered when an unsuitable drug was administered based on later in vitro susceptibility results or in patients without antifungal treatment [15]. Crude mortality was recorded 30 days following the onset of the *Candida* BSI episode.

### Statistical Analysis

IBM SPSS Statistics version 25 was used for the analysis of the data. Numerical data were provided as mean and standard deviation, or median and range, as relevant. For qualitative data, frequency and percentage were employed. The *χ*^2^ test, or Fisher's exact test, was used to analyze the relationship between qualitative variables. The Mann–Whitney test compared two groups of non-normally distributed quantitative data. The *κ* test assessed the agreement between the two diagnostic methods. Values between 1 and 20% denote slight agreement, 21% and 40% fair agreement, 41% and 60% moderate agreement, 61% and 80% high agreement, and 81% and 100% approximately complete agreement. Clinically relevant variables with *P*-values less than 0.2 in univariate analysis were utilized in multivariable logistic regression analysis to identify predictive and associated factors for NAC BSIs. Related variables were each tested in a separate regression model to avoid multicollinearity. The goodness-of-fit of those models was evaluated by the Hosmer–Lemeshow test. A non-significant test of more than 0.05 indicates a good fit. Then we select the appropriate model for our research accordingly (other models are not shown). A *P*-value < 0.05 was considered significant.

## Results

A total of 1701 BSIs were detected during a 1-year study. Of these, 53 episodes of candidemia account for 3.1% of all positive blood cultures. We included 30 patients in our analysis after excluding outpatients, duplicate cases, and patients with missing or incomplete data. The baseline characteristics of those 30 cancer patients with hematological and solid organ tumors are shown (Table 1). Previous exposure to antimicrobials was observed in most patients (96.7%). Other risk factors such as prolonged hospital stay (≥ 7 days), ICU admission, chemotherapy, and surgical procedures were all revealed in our patients (73.3%, 50.0%, 66.7%, and 30.0%, respectively).

**Table 1:** Baseline characteristics of cancer patients with candidemia at the NCI

Variables	Total (*n* = 30)	Patients with		*P*-value
		Hematological cancer (*n* = 15)	Solid organ tumors (*n* = 15)	
	No. of cases (%) or median and range			
Age (years)	30 (1–80)	19 (1–80)	44 (2–75)	0.290
< 18	13 (43.3)	7 (46.7)	6 (40.0)	0.713
18–50	8 (26.7)	6 (40.0)	2 (13.3)	0.215
> 50	9 (30.0)	2 (13.3)	7 (46.7)	0.109
Gender				
Male	19 (63.3)	11 (73.3)	8 (53.3)	0.256
Female	11 (36.7)	4 (26.7)	7 (46.7)	
Duration of hospital stay^a^ (days)	15 (3–62)	19 (3–62)	9 (3–20)	**0.027**
Duration of hospital stay^a^				
≥ 7 days	22 (73.3)	12 (80.0)	10 (66.7)	0.682
< 7 days	8 (26.7)	3 (20.0)	5 (33.3)	
ICU admission				
Yes	15 (50.0)	5 (33.3)	10 (66.7)	0.068
No	15 (50.0)	10 (66.7)	5 (33.3)	
Chemotherapy				
Yes	20 (66.7)	15 (100.0)	5 (33.3)	** < 0.001**
No	10 (33.3)	0 (0.0)	10 (66.7)	
Surgical procedures				
Yes	9 (30.0)	0 (0.0)	9 (60.0)	**0.001**
No	21 (70.0)	15 (100.0)	6 (40.0)	
Prophylaxis antifungal				
Yes	8 (26.7)	8 (53.3)	0 (0.0)	**0.002**
No	22 (73.3)	7 (46.7)	15 (100.0)	
Combined antifungal therapy				
Yes	5 (16.7)	5 (33.3)	0 (0.0)	**0.042**
No	25 (83.3)	10 (66.7)	15 (100.0)	
Inappropriate antifungal therapy				
Yes	17 (56.7)	4 (26.7)	13 (86.7)	**0.001**
No	13 (43.3)	11 (73.3)	2 (13.3)	
Duration of episode (days)	12 (1–50)	26 (3–50)	7 (1–24)	**0.009**
Laboratory findings				
Total leukocytic count (cell/μl)	3000 (36–77,000)	1200 (36–11,000)	16,000 (140–77,000)	** < 0.001**
Absolute neutrophil count (cell/μl)	791 (0–71,610)	80 (0–3034)	11,900 (9.8–71,610)	** < 0.001**
30-day mortality				
Died	11 (36.7)	3 (20.0)	8 (53.3)	0.058
Alive	19 (63.3)	12 (80.0)	7 (46.7)	

Statistically significant *p*-values less than 0.05 are in boldface

^a^Refers to the period of hospitalization before the onset of infection

Candidemia has been detected equally frequently in hematological and solid organ tumor patients. Most hematological cancers (*n* = 12, 80.0%) were acute leukemia, whereas solid organ tumors were mainly in the genitourinary tract, followed by the central nervous system and the gastrointestinal tract (40.0%, 33.3%, and 26.7%, respectively). Patients with hematological malignancies had longer infection episodes, and longer hospital stays before the infection (*P* = 0.009 and 0.027, respectively). Those patients were significantly more likely to have taken prophylactics and been treated with a combination of antifungals (*P* = 0.002 and 0.042, respectively). The total 30-day post-candidemia mortality rate was 36.7%, with patients who had solid organ tumors dying at a greater rate than those with hematological malignancies (53.3% versus 20.0%, *P* = 0.058).

**Fig. 1:**
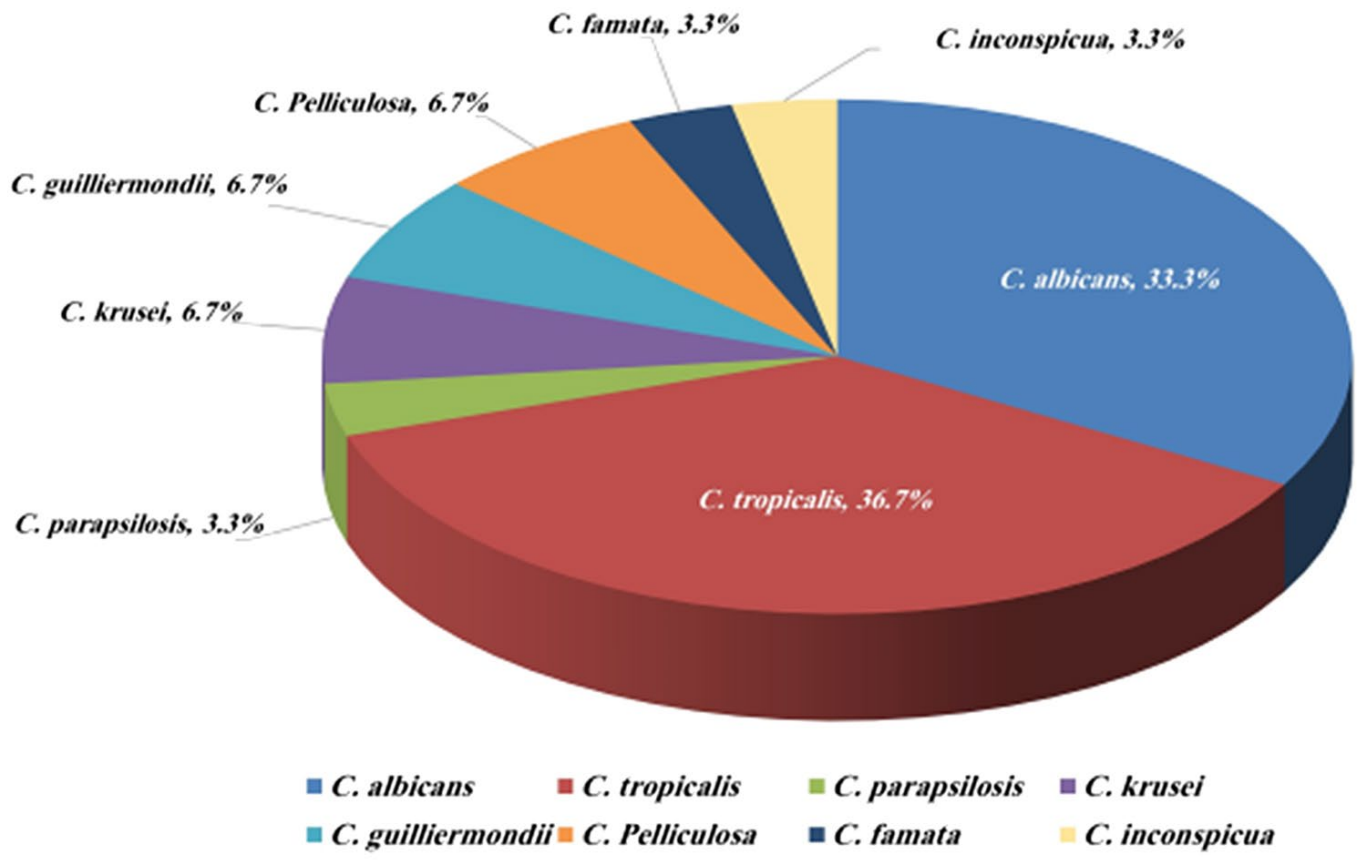
Frequency of *Candida* species identified by Vitek 2 causing bloodstream infections in cancer patients at the National Cancer Institute**.** The percentages of all identified species are shown. *Candida tropicalis* was the most prevalent species (*n* = 11/30, 36.7%), followed by *C. albicans* (*n* = 10, 33.3%). Less isolated were *C. krusei*, *C. guilliermondii*, *C. pelliculosa* (each with 2 isolates, 6.7%), *C. parapsilosis*, *C. famata*, and *C. inconspicua* (each with 1 isolate, 3.3%)

The species identified by Vitek 2 that cause candidemia among cancer patients are demonstrated (Fig. 1). *C. tropicalis* was the most common species (*n* = 11, 36.7%), while *C. glabrata* was not detected in all the 30 cancer patients' blood cultures. Coexisting bacterial infections were found in 56.7% of patients, with higher rates in patients with solid organ tumors than in patients with hematological malignancies (66.7% versus 46.7%, *P* = 0.269). Interestingly, 40.0% of patients had multifocal *Candida* infections in other body sites. These multifocal infections were significantly associated with solid organ tumor patients (60.0% versus 20.0%, *P* = 0.025). The results of sn-PCR and CHROMagar revealed a significant agreement with those of Vitek 2 (Table 2), with the overall concordance in the identification of *Candida* species found to be 77.5% and 86.0% (*P* < 0.001), respectively.

**Table 2:** Frequency of isolation of different *Candida* species by molecular, automated, and phenotypic methods

*Candida* species	No., % of isolates identified by			Agreement with Vitek 2 (%, *P*-value)	
	Vitek 2	PCR	CHROMagar	PCR	CHROMagar
*C. albicans*	10, 33.3	8, 26.7	9, 30.0	84.2, < 0.001	92.3, < 0.001
*C. tropicalis*	11, 36.7	9, 30.0	10, 33.3	85.1, < 0.001	92.7, < 0.001
*C. parapsilosis*	1, 3.3	3, 10.0	#	47.4, 0.002	NC
*C. krusei*	2, 6.7	2, 6.7	2, 6.7	100.0, < 0.001	100.0, < 0.001
*C. guilliermondii*	2, 6.7	*	#	NC	NC
*C. pelliculosa*	2, 6.7	*	#	NC	NC
*C. famata*	1, 3.3	*	#	NC	NC
*C. inconspicua*	1, 3.3	*	#	NC	NC

*NC* non-calculable

*Unidentified species by PCR (*Candida* species other than *C. albicans*, *C. tropicalis*, *C. parapsilosis*, *C. krusei,* and *C. glabrata*) as their primers were not included in the PCR amplification step, # Unidentified species by CHROMagar

A separate analysis was performed to identify patients' demographic data, clinical characteristics, underlying disease, risk factors, patient outcomes, and microbiological findings associated with NAC species isolated from blood cultures of cancer patients with candidemia (Table 3). NAC BSI was more common in children, male patients, and patients with hematological malignancies and was associated with increased coexistent bacteremia (*P* = 0.303, 0.289, 0.130, and 0.603, respectively). On the other hand, *C. albicans* was more commonly associated with surgical procedures, prolonged hospital stays, and the presence of multifocal *Candida* infection, and infected patients had a higher mortality rate (*P* = 0.017, 0.562, 0.122, and 0.069, respectively).

**Table 3:** Univariate analysis of risk factors, clinical and microbiological characteristics associated with non-albicans *Candida* species infections in cancer patients with candidemia

Risk factors	No. of cases (%)		Odds ratio	95% CI	*P*-value
	Non-albicans *Candida* (*n* = 20)	*C. albicans* (*n* = 10)			
Age					
< 18 years (*n* = 13)	10 (50.0)	3 (30.0)	2.333	0.466–11.693	0.303
18–50 years (*n* = 8)	5 (25.0)	3 (30.0)	0.778	0.144–4.212	0.771
˃50 years (*n* = 9)	5 (25.0)	4 (40.0)	0.500	0.099–2.527	0.402
Male sex (*n* = 19)	14 (70.0)	5 (50.0)	2.333	0.488–11.167	0.289
Type of cancer					
Hematological malignancies (*n* = 15)	12 (60.0)	3 (30.0)	3.500	0.692–17.714	0.130
Gastrointestinal tract cancer (*n* = 4)	0 (0.0)	4 (40.0)	**NC**	**NC**	**0.002**
Genitourinary tract cancer (*n* = 6)	4 (20.0)	2 (20.0)	1.000	0.150–6.671	1.000
Central nervous system cancer (*n* = 5)	4 (20.0)	1 (10.0)	2.250	0.217–23.324	0.497
Prolonged hospital stay ≥ 7 days (*n* = 22)	14 (70.0)	8 (80.0)	0.583	0.094–3.603	0.562
ICU admission (*n* = 15)	10 (50.0)	5 (50.0)	1.000	0.219–4.564	1.000
Chemotherapy (*n* = 20)	16 (80.0)	4 (40.0)	**6.000**	**1.125–31.989**	**0.036**
Surgical procedures (*n* = 9)	3 (15.0)	6 (60.0)	**0.118**	**0.020–0.686**	**0.017**
Prophylaxis antifungal (*n* = 8)	6 (30.0)	2 (20.0)	1.714	0.278–10.589	0.562
Inappropriate antifungal therapy (*n* = 17)	10 (50.0)	7 (70.0)	0.429	0.086–2.148	0.303
Leucopenia (*n* = 16)	14 (70.0)	2 (20.0)	**9.333**	**1.511–57.654**	**0.016**
Neutropenia (*n* = 16)	13 (65.0)	3 (30.0)	4.333	0.845–22.230	0.079
Coexistent bacterial infection (*n* = 17)	12 (60.0)	5 (50.0)	1.500	0.325–6.918	0.603
Multifocal *Candida* infections (*n* = 12)	6 (30.0)	6 (60.0)	0.286	0.059–1.395	0.122
30-day mortality (*n* = 11)	5 (25.0)	6 (60.0)	0.222	0.044–1.123	0.069

Statistically significant *P*-values less than 0.05 are in boldface

*ICU* intensive care unit, *CI* confidence interval, *NC* non-calculable

Chemotherapy and leucopenia were significantly associated with an increased risk of BSIs caused by NAC species, with *P*-values of 0.036 and 0.016, respectively. Furthermore, patients with NAC candidemia had significantly lower TLC and ANC than *C. albicans* candidemia patients, with median and *P*-values of 1500 versus 17,000, *P* = 0.001, and 288 versus 12,565, *P* = 0.022, respectively. Leucopenia was identified as an independent risk factor for NAC candidemia in a multivariable logistic regression analysis (*P*-value = 0.048, Table 4).

**Table 4:** Multivariable logistic analysis of risk factors, clinical and microbiological characteristics associated with non-albicans *Candida* species infections in cancer patients with candidemia

Variables	Adjusted odds ratio	95% Confidence interval for adjusted odds ratio		*P*-value
		Lower limit	Upper limit	
Multifocal infections	0.649	0.067	6.257	0.708
Presence of leucopenia	10.065	1.018	99.529	**0.048**
Unfavorable outcome	0.260	0.039	1.735	0.164

Hosmer and Lemeshow Test: *P*-value = 0.951, statistically significant *P*-values less than 0.05 are in boldface

In vitro antifungal susceptibility results and MIC values of *Candida* species isolates are presented (Table 5). According to CLSI (2022) and as shown in Table 5, 6 isolates of *Candida* species in our study have no CBPs established yet, including 2 *C. guilliermondii*, 2 *C. pelliculosa*, 1 *C. famata*, and 1 *C. inconspicua*. Four (16.7%) of the 24 isolated *Candida* species with established CBPs were resistant to in vitro voriconazole, including 2 *C. tropicalis*, 1 *C. albicans*, and 1 *C. parapsilosis*, with a median MIC of 0.125 μg/ml ranging from 0.002 to 32 μg/ml. NAC species exhibited a greater voriconazole resistance rate, median MIC value, and range than *C. albicans* [21.4% versus 10.0% and 0.220 (0.002–32) versus 0.048 (0.002–0.750) μg/ml with a *P*-value of 0.047].

**Table 5:** Antifungal susceptibility results and MIC values of *Candida* species isolates in cancer patients with candidemia

*Candida* species	No of isolates (%)	Drug	MIC (μg/ml)			No. (%) of isolates by CBPs			No. (%) of isolates by ECVs	
			Range	MIC_50_	MIC_90_	S	I/SDD	R	WT	NWT
*C. albicans*	10 (33.3)	VRC	0.002–0.75	0.032	0.25	8 (80.0)	1 (10.0)	1 (10.0)	5 (50.0)	5 (50.0)
	10 (33.3)	FLC	1.5– > 256	3	> 256	4 (40.0)	1 (10.0)	5 (50.0)	0 (0.0)	10 (100.0)
*C. tropicalis*	11 (36.7)	VRC	0.006–24	0.25	8	5 (45.5)	4 (36.3)	2 (18.2)	5 (45.5)	6 (54.5)
	11 (36.7)	FLC	1– > 256	6	> 256	5 (45.5)	0 (0.0)	6 (54.5)	1 (9.1)	10 (90.9)
*C. parapsilosis*	1 (3.3)	VRC	32	ND	ND	0 (0.0)	0 (0.0)	1 (100.0)	–	–
	1 (3.3)	FLC	> 256	ND	ND	0 (0.0)	0 (0.0)	1 (100.0)	0 (0.0)	1 (100.0)
*C. krusei*	2 (6.7)	VRC	0.012–1	0.012	1	1 (50.0)	1 (50.0)	0 (0.0)	1 (50.0)	1 (50.0)
	2 (6.7)	FLC	> 256	> 256	> 256	–	–	IR	–	–
*C. guilliermondii*	2 (6.7)	VRC	0.19–32	0.19	32	–	–	–	–	–
	2 (6.7)	FLC	> 256	> 256	> 256	–	–	–	0 (0.0)	2 (100.0)
*C. pelliculosa*	2 (6.7)	VRC	0.006–0.064	0.006	0.064	–	–	–	2 (100.0)	0 (0.0)
	2 (6.7)	FLC	1.5–2	1.5	2	–	–	–	2 (100.0)	0 (0.0)
*C. famata*	1 (3.3)	VRC	0.002	ND	ND	–	–	–	–	–
	1 (3.3)	FLC	0.19	ND	ND	–	–	–	–	–
*C. inconspicua*	1 (3.3)	VRC	24	ND	ND	–	–	–	–	–
	1 (3.3)	FLC	> 256	ND	ND	–	–	–	–	–

Minimum inhibitory concentration (MIC) is described as a MIC range, MIC_50_, and MIC_90_. Only species with more than one isolate are calculated for MIC_50_ and MIC_90_. No clinical breakpoints (CBPs) or epidemiologic cutoff values (ECVs) for *C. krusei* and fluconazole because of the species' IR. ECVs were used for the classification of isolates into wild-type (WT) and non-WT (NWT) azole susceptibility phenotypes in the absence of CBPs for species according to CLSI, M27M44S-Ed3. No CBPs nor ECVs were found for voriconazole and *C. guilliermondii*, *C. famata*, or *C. inconspicua*, or for fluconazole and *C. famata* or *C. Inconspicua*

*VRC* voriconazole, *FLC* fluconazole, *S* sensitive, *I* intermediate, *SDD* susceptible dose-dependent, *R* resistant, *ND* not determined, *IR* intrinsic resistance

**Fig. 2:**
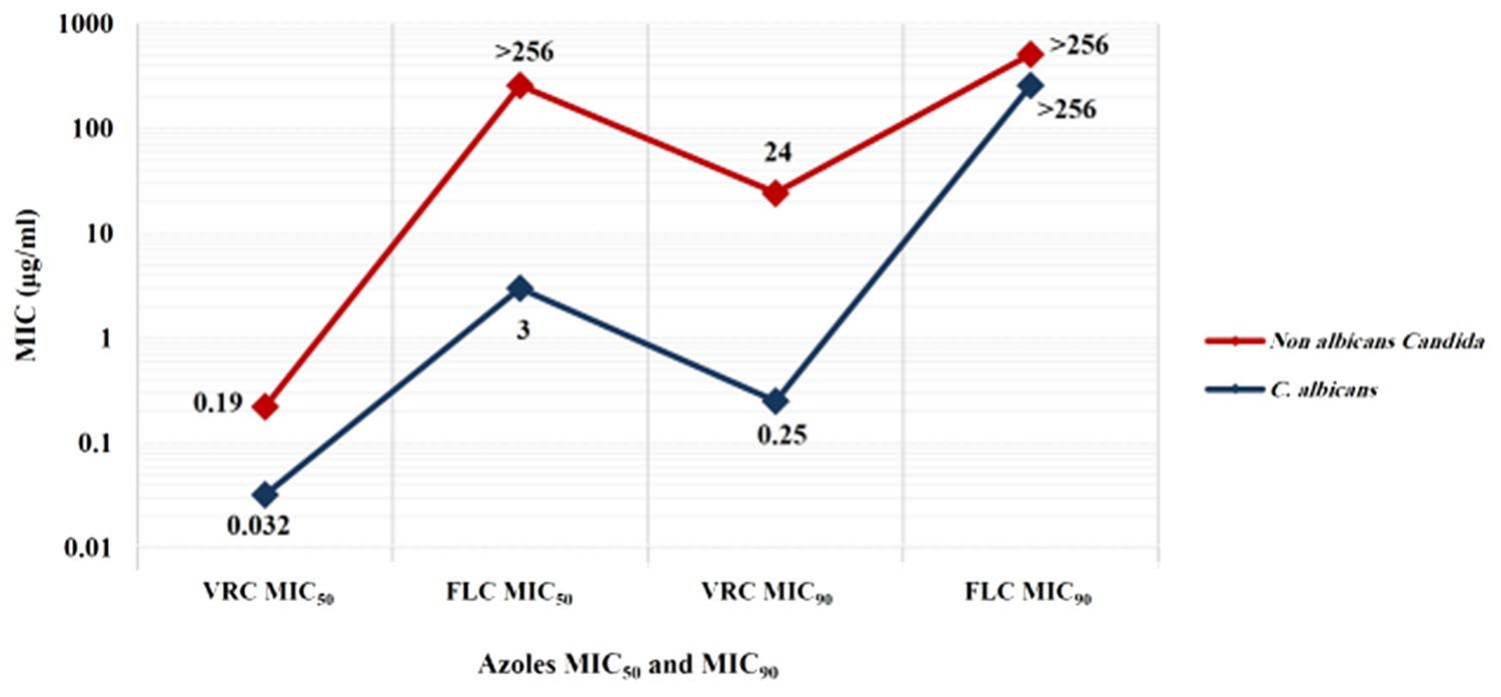
Voriconazole and fluconazole MIC_50_ and MIC_90_ in *Candida albicans* and non-albicans *Candida* species causing bloodstream infections in cancer patients at the National Cancer Institute. NAC species exhibited higher MIC_50_ and MIC_90_ values for voriconazole and fluconazole than *C. albicans*. Voriconazole MIC_50_ and MIC_90_ values for NAC and *C. albicans* species were 0.19 versus 0.032 and 24 versus 0.25 μg/ml, respectively; fluconazole MIC_50_ was > 256 versus 3 μg/ml, respectively, and MIC_90_ was > 256 μg/ml in both NAC and *C. albicans* species. The MIC_50_ and MIC_90_ represent the lowest concentration of the antifungal agent that inhibits 50% and 90% of the isolates, and based on the formulae, MIC_50_ = number of isolates × 0.5 and MIC_90_ = number of isolates × 0.9, respectively. *MIC* minimum inhibitory concentration, *NAC* non-albicans *Candida*

The overall resistance rate for fluconazole was high (14 isolates, 58.3%), including 5 *C. albicans*, 6 *C. tropicalis*, and 1 *C. parapsilosis*, besides 2 *C. krusei* strains that have an inherent resistance to fluconazole. Despite a high median MIC for fluconazole against all *Candida* species of > 256 ranging from 0.190 to > 256 μg/ml, *C. albicans* had a lower resistance rate, median MIC value, and range than NAC species (50.0% versus 64.3%, 129 (1.5–> 256) versus > 256 (0.190–> 256) μg/ml with *P* = 0.981). Furthermore, the MIC_50_ and MIC_90_ of voriconazole and fluconazole in NAC species were higher than in *C. albicans* (NAC and *C. albicans* species voriconazole MIC_50_ and MIC_90_ were 0.19 versus 0.032 and 24 versus 0.25 μg/ml, respectively; fluconazole MIC_50_ was > 256 versus 3 μg/ml, respectively, and MIC_90_ of > 256 μg/ml in both NAC and *C. albicans* species) (Fig. 2). However, the median MIC for fluconazole and voriconazole did not differ statistically between hematological and solid tumor patients (*P* = 0.665 and 0.708, respectively).

## Discussion

Candidemia is one of the most common invasive fungal infections in immunocompromised cancer patients. *Candida* was the third-leading cause of BSIs in the United States and the seventh cause in Europe [6, 16]. In the current study**,** *Candida* species isolates represented 3.1% of all positive blood cultures. This isolation rate was parallel to the results of Lim et al., where 3.0% of all positive blood cultures were fungal infections, with the majority being *Candida* species (95%) [17]. Despite the dominance of *C. albicans* as the pathogenic *Candida* species, a global rise of NAC species has been reported in recent decades. Studying local trends in *Candida* species distribution and antifungal susceptibility is essential since they differ considerably among countries or institutions and are influenced by patients' underlying conditions [2].

*Candida tropicalis* was the most common species in our patients, followed by *C. albicans* (36.7 and 33.3%). But *C. krusei*, *C. guilliermondii*, *C. pelliculosa*, *C. parapsilosis*, *C. famata*, and *C. inconspicua* were less isolated. *Candida* species have shown variation in different geographical areas, with more reported *C. albicans* in some regions [1, 2, 18, 19], compared to *C. tropicalis* across Africa, Asia, and Latin America [20–22]. However, a significant trend in *Candida* species distribution among patients having certain underlying disorders has been noticed. Similar to our results, a higher proportion of *C. tropicalis* and *C. krusei* was consistently recorded among candidemia patients with hematological malignancies [23, 24].

Some studies have implicated broad-spectrum antibiotics and immunosuppression as risk factors for candidemia caused by certain non-albicans species, such as *C. famata* and *C. pelliculosa* [25, 26]. Interestingly, both previously stated species were identified in three of our pediatric patients treated with chemotherapy. *C. famata* was also detected in children with cancer in a study by Vasileiou et al. [27]. Although *C. pelliculosa* was documented primarily in neonates, it has also been reported at a lower frequency in other age groups, consistent with our findings [28]. The increased detection of these species recently, particularly in high-risk patients, necessitates further studies, as their significance is not yet fully understood.

The worldwide rise in azole resistance has caused severe therapeutic challenges. This rise may be because of the widespread use of azoles, particularly in low-income countries, and their fungistatic mode of action against *Candida* [2, 5]. A prior study was conducted on candidemia, including eighteen healthcare facilities. Fluconazole resistance was significantly linked with yearly inpatient days (hospital volume) (*P* < 0.001) and cancer and transplantation settings (*P* < 0.001) and was ten times more prevalent in larger institutions (*P* < 0.001) [29]. Additionally, prior azole exposure and an inadequate dose or duration of treatment also contributed to such high azole antifungal resistance, particularly in NAC [30].

Fluconazole and voriconazole were the most frequently utilized antifungals in our patients. Such azoles were used in 87.5% of prophylactic and 73.3% of antifungal treatments. After we excluded the six *Candida* isolates with no established CBPs, fluconazole and voriconazole resistance accounted for 58.3% and 16.7% of total *Candida* BSIs, respectively. All species showed high resistance and MIC values to azoles, notably fluconazole, which showed higher resistance rates in NAC than in *C. albicans* (64.3% versus 50.0%). These results agreed with an Egyptian study that found high fluconazole resistance rates from NAC and *C. albicans* species causing BSIs in pediatric patients at Cairo University pediatric hospitals (44.0% and 38.9%, respectively) [31]. Another Egyptian study also revealed elevated rates of resistance to voriconazole and fluconazole in all species, including *C. albicans*, *C. krusei*, *C. glabrata*, *C. tropicalis*, and *C. parapsilosis* (38.4% and 38.5%; 21.5% and 100.0%; 100.0% and 40.0%; 12.5% and 25.0%; and 10.0% and 20.0%, respectively) in ICU patients at Ain Shams University Hospital [32]. A previous study from the last decade found lower fluconazole resistance of 18.9% and 11.4% in NAC and *C. albicans* species, respectively. It was conducted on pediatric candidemia patients at Egypt's Mansoura University Children's Hospital [33]. Therefore, it is evident that azole resistance in candidemia is increasing in Egyptian hospitals.

Several studies from other countries also found high MICs and resistance rates for azoles, mainly for fluconazole. However, these high values were detected only in NAC species and at lower rates. According to a study conducted in China, 50.0% and 56.5% of *C. tropicalis* isolates were resistant to fluconazole and voriconazole, respectively [30]. Another Chinese study also revealed elevated fluconazole resistance rates among *C. tropicalis* (47.6%) as well as in uncommon *Candida* species (45.5%) [2]. Additionally, studies in Algeria [34], Saudi Arabia [7], and South Africa [18] showed high resistance rates to fluconazole in NAC.

Epidemiologic cutoff values can identify *Candida* isolates with and without acquired resistance based on their phenotypes. An organism with a MIC greater than the ECV is assumed to have mutational resistance (non-WT). ECVs should not replace the published CBPs in clinical practice since they do not categorize isolates into sensitive and resistant (treatable and untreatable) isolates. However, it can guide physicians in making clinical decisions when CBPs are unavailable [35]. Furthermore, there is no direct relationship between CBPs and ECVs. Unlike CBPs, they may be used in surveillance to detect trends toward increased resistance within a species and to determine empirical therapy by detecting local susceptibility patterns. ECVs can identify whether a species, although still sensitive, is not typical or WT [12].

Our *Candida* isolates showed increased rates of non-WT phenotypes for azoles, with MIC values higher than ECVs. Fluconazole and voriconazole non-WT phenotypes were identified in 88.5% and 48.0% of all isolated *Candida* species. Furthermore, we found severe azole cross-resistance among our isolates. All voriconazole-resistant *Candida* isolates were fluconazole-resistant (100%). In addition, all isolates that showed non-WT phenotypes for voriconazole were fluconazole non-WT phenotypes (100%). Most of our isolates showed elevated MIC values for azoles compared to published studies from other countries. Non-WT phenotypes of fluconazole and voriconazole were observed in 61.9% and 33.3% of *C. tropicalis*, 60.0% and 45.0% of *C. glabrata*, and 20.8% and 16.7% of *C. albicans* in Korea [36]; 17% and 24% of *C. guilliermondii*, 16% and 7% of *C. lusitaniae*, and 8% and 17% of *C. kefyr* in the USA [37]; 36.9% and 47.4% of *C. tropicalis*, and 5.6% and 5.6% of *C. albicans* in Algeria [34]. Consistent with our results, cross-resistance among azole antifungals was recorded in many studies [4, 30].

Cancer patients are a unique population of patients subjected to multiple courses of antibiotics and antifungals whenever they manifest a persistent fever that does not respond to antibiotics. Thus, they are more at risk of developing resistance. Because fluconazole has been linked to high rates of resistance in many parts of the world, it should be used cautiously in high-risk patients who are unsure of their response. Furthermore, based on the high resistance rates of fluconazole shown in our study and other Egyptian studies, a stewardship program is needed to preserve voriconazole so as not to be subjected to increasing resistance with misuse.

In this study, although sn-PCR and CHROMagar results showed a significant agreement with those of Vitek 2, sn-PCR and CHROMagar could not identify 26.6% and 30.0% of *Candida* to the species level. Therefore, conventional techniques are insufficient for *Candida* species identification, which causes invasive candidiasis. Broader spectrum techniques, such as matrix-assisted laser desorption/ionization-time of flight (MALDI-TOF), more advanced ones, such as PCR-restriction fragment length polymorphism (PCR-RFLP), and sequencing or combined methods are recommended whenever applicable, particularly in patients at high risk for candidemia.

Broad-spectrum antibiotics, prolonged hospital stays, ICU, chemotherapy, neutropenia, recent surgeries, coexisting bacteremia, and multifocal *Candida* infections were all observed as risk factors in our patients (73.3%, 50.0%, 66.7%, 53.3%, 20.0%, 56.7%, and 40.0%, respectively). Several studies have also revealed these variables as significant factors in the development of candidemia [7, 22, 30, 38, 39]. Even though hematological patients are more at risk for candidemia than those with solid tumors, both have been predictors of candidemia [40]. We revealed *Candida* BSIs in patients with hematologic and solid tumors equally. Surprisingly, in studies by Zheng et al. and Liu et al., solid cancers were more prevalent than hematological malignancies (30.0% versus 12.2% and 23.6% versus 4.0%, respectively) [1, 30]. These findings emphasize the risk of candidemia in both patients with solid organ and hematological cancers. Thus, increasing clinician awareness of risk factors and appropriate treatment strategies for invasive *Candida* infections is critical for optimal patient care. Candidemia caused by NAC species was significantly associated with chemotherapy and leucopenia. On the other hand, in adult patients, surgeries, prolonged hospitalization, and multifocal infections were predictive factors linked to candidemia caused by *C. albicans*. In multivariable analysis, leucopenia was the independent factor associated with an increased risk of NAC BSIs. Likewise, hematological malignancies, chemotherapy, leucopenia, and neutropenia were identified by other studies as increasing the risk of NAC, whereas surgeries, old age, and catheters increase the risk of *C. albicans* BSIs [2, 20, 30].

The current study has some limitations. Although the NCI treats cancer patients from all over the country, our findings were limited to a single institute. Our study's sample size was relatively small, which may impact the risk factor analysis. Moreover, since *C. auris* is rare in our country and the tested species account for more than 90% of cases, we focused on the prevalence of common *Candida* species rather than *C. auris* among our patients. A recent study in our country failed to detect *C. auris* in 414 candidiasis-causing isolates [41]. In addition, we did not analyze clonality within the same species to assess strain-relatedness using pulsed-field gel electrophoresis (PFGE) or multilocus sequence typing (MLST). Furthermore, we did not test for susceptibility to echinocandin or amphotericin B as we did for azoles, our nation's most widely used antifungal.

## Conclusion

We revealed the predominance of NAC-causing candidemia in oncology patients, with *C. tropicalis* being the most prevalent species. NAC candidemia was significantly linked to chemotherapy and leucopenia. We believe that chemotherapy-induced leucopenia and neutropenia in cancer patients are strongly linked to NAC BSIs rather than an underlying tumor, such as hematological malignancies. Our findings demonstrated an increased reduction in fluconazole and voriconazole susceptibility, particularly in NAC species. The general critical condition of cancer patients and numerous risk factors, such as prolonged hospitalization, all contributed to these susceptibility trends. Furthermore, the extensive use of azoles at our institute enhanced the selection pressure for resistance. The high fluconazole resistance rates and the increasing prevalence of NAC suggest a potential decrease in therapeutic efficacy and point to the necessity of stewardship programs to preserve voriconazole efficacy. Moreover, this alarming rise in azole resistance rates and elevated MIC values highlights the importance of physician awareness, early detection, and continued future surveillance of candidemia, especially in this critically ill group of patients. Moreover, investigating the underlying molecular mechanisms of azole resistance is highly advised, as this may improve the understanding of this critical clinical situation.

### Supplementary Information

Below is the link to the electronic supplementary material.

Supplementary file1 (DOCX 369 KB)

## Data Availability

The data collected and evaluated during the current study are accessible upon reasonable request from the corresponding author.
