# Supplementary material for: Alarming Increase of Azole-Resistant Candida Causing Blood Stream Infections in Oncology Patients in Egypt
Source: Curr Microbiol. 2023 Oct 5;80(11):362. doi: 10.1007/s00284-023-03468-w (PMC10556164; doi:10.1007/s00284-023-03468-w)
Supplement: Supplementary file 1 — Supplementary file1 (DOCX 369 KB) [file 284_2023_3468_MOESM1_ESM.docx]

**Journal name**

**Current Microbiology**

**Article title**

**Alarming Increase of Azole-Resistant *Candida* Causing Blood Stream Infections in Oncology Patients** **in Egypt**

Hadir A. El-Mahallawy^1^, Nesma E. Abdelfattah^1^, Mona A. Wassef^2^, Rasha M. Abdel-Hamid^1*^

^1^Clinical Pathology Department, National Cancer Institute, Cairo University, Cairo, Egypt

^2^Clinical & Chemical Pathology Department, Faculty of Medicine, Cairo University, Cairo, Egypt

***Corresponding author**

Dr. Rasha Mahmoud Abdel-Hamid, MD., PhD.

Clinical Pathology Department, NCI, Cairo University, Cairo, Egypt

Tel: +201007063322

Email: [rasha.elgyar@nci.cu.edu.eg](about:blank)

ORCID number: 0000-0002-2177-6153

**Supplementary materials**

**Multiplex semi-nested polymerase chain reaction (sn-PCR)**

Multiplex sn-PCR was used for *Candida* species identification using five *Candida* species primers (*C. albicans, C. tropicalis, C. glabrata,* *C. krusei*, and *C. parapsilosis*).

**DNA extraction:**

The QIAamp DNA Mini Kits (QIAGEN, Hilden, Germany) were used according to the manufacturer's instructions for DNA extraction. The quantity and quality of DNA were evaluated using a Nanodrop spectrophotometer (NanoDrop ND1000, NanoDrop, USA), and purity was assessed using a 260/280 ratio. DNA concentration of 20-120ng/ul was accepted for further PCR steps. DNA purity of 260/280 ratio range (1.6-1.9) was accepted for further PCR steps.

**PCR amplification:**

The PCR amplification first used the fungus-specific universal oligonucleotides internal transcribed spacer 1 and 4 (ITS1 and ITS4) as outer primers. Then the inner primers of *C. krusei*, *C. glabrata*, *C. albicans*, *C. parapsilosis*, and *C. tropicalis* were used in the second run. The sequences of the primers used are shown in Table A1 [8].

The first round of amplification was performed in a 25 μl reaction mixture containing 12.5 micron PCR master mix (Dream Taq Green PCR Master Mix, Thermo Scientific, Lithuania), 50 ng of purified DNA serving as the DNA template, 0.2 µM each primer (ITS1 and ITS4), and water (nuclease-free) to complete the volume (to 25 μl). The PCR cycling conditions were as follows: an initial denaturation phase of 5 minutes at 95°C followed by 35 cycles of 45 seconds at 95°C, 45 seconds at 50°C, and 45 seconds at 72°C, with a final extension of 5 minutes at 72°C. The amplification reactions were carried out in a DNA-Thermal cycler (Biometra).

The second round of amplifications was carried out in two separate assays: assay 1, containing primers CTR, CALB, and CGL at concentrations of 0.12 μM, 0.2 μM, and 0.3 μM, respectively. Assay 2 containing primer CKR and CPAR at 0.2 μM and 0.15 μM concentrations, respectively. In both assays, 2 μl of a 1:100 dilution of the ITS PCR product was used as the DNA template. This template was mixed with the inner primers and 5% dimethyl sulfoxide to fresh reaction mixtures in a total volume of 25 μl. The PCR amplification conditions were: an initial denaturation step of 5 minutes at 95°C, 10 cycles of 45 seconds at 95°C, 45 seconds at 67-58°C, and 45 seconds at 72°C followed by 20 cycles of 45 seconds at 95°C, 45 seconds at 58°C, and 45 seconds at 72°C, with a final extension of 5 minutes at 72°C.

The nested PCR outputs were then detected by 2% agarose gel electrophoresis and visualized using an ultraviolet transilluminator (Biometra). The amplicon sizes are reported in Table A1 [8].

**Table A1 Primers employed in the multiplex nested PCR amplifications [8]**

| **Primers** | **Sequences (5' → 3')** | **Amplicons** |
| --- | --- | --- |
| ITS 1/4 | F-TCCGTAGGTGAACCTGCGG  R-TCCTCCGCTTATTGATATGC | variable |
| CALB | F-TTTATCAACTTGTCACACCAGA  R-ATCCCGCCTTACCACTACCG | 272 bp |
| CPAR | F-GCCAGAGATTAAACTCAACCAA  R-CCTATCCATTAGTTTATACTCCGC | 297 bp |
| CTR | F-CAATCCTACCGCCAGAGGTTAT  R-TGGCCACTAGCAAAATAAGCGT | 357 bp |
| CKR | F-ACTACACTGCGTGAGCGGAA  R-AAAAAGTCTAGTTCGCTCGG | 362 bp |
| CGL | F-TTATCACACGACTCGACACT  R-CCCACATACTGATATGGCCTACAA | 423 bp |

ITS: internal transcribed spacers, F: forward, R: reverse, CALB: *C. albicans*, CGL: *C. glabrata*, CTR: *C. tropicalis*, CKR: *C. krusei*, CPAR: *C. parapsilosis* complex, bp: base pairs


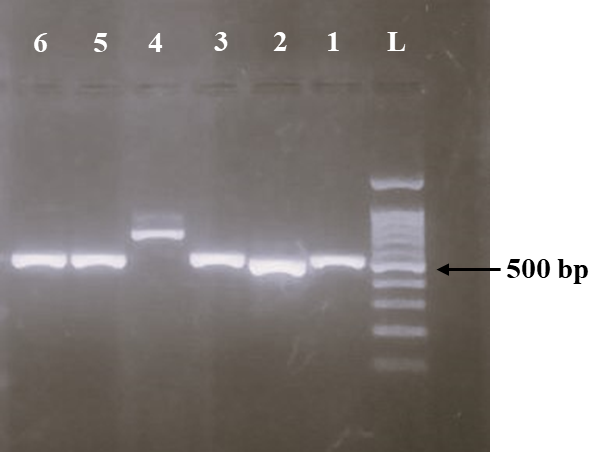


**Figure A1** Agarose gel displays the products of PCR amplification using the outer primers ITS1 and ITS4. Lane L:100 bp DNA ladder marker (Thermo Scientific, Lithuania), Lanes 1 to 6: ITS1/4 positive (variable amplicon sizes). ITS: internal transcribed spacers


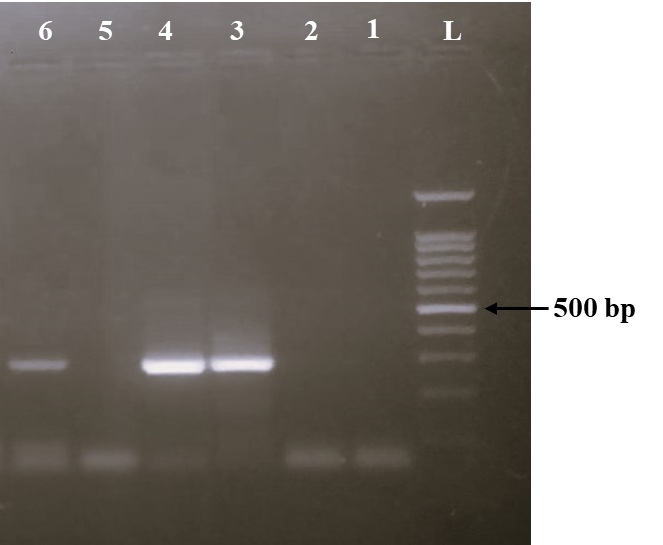


**Figure A2** Agarose gel displays the nested multiplex products of PCR amplification using the inner primers (CKR and CPAR) for assay 2. Lane L:100 bp DNA ladder marker (Thermo Scientific, Lithuania), Lanes 3, 4, and 6: *C. parapsilosis* complex (297 bp), Lanes 1,2 and 5: negative for *C. parapsilosis* complex (297 bp) and *C. krusei* (362 bp). CKR: *C. krusei*, CPAR: *C. parapsilosis* complex
